# Effect of Pretransplant Use of Calcimimetic on Parathyroid Function after Renal Transplantation

**DOI:** 10.1155/2021/1999777

**Published:** 2021-09-27

**Authors:** Kanako Bokuda, Satoshi Morimoto, Yasufumi Seki, Noriyoshi Takano, Atsuhiro Ichihara

**Affiliations:** Department of Endocrinology and Hypertension, Tokyo Women's Medical University, Tokyo, Japan

## Abstract

**Objective:**

Persistence of hyperparathyroidism (HPT) after renal transplantation leads to undesirable outcomes such as increase in cardiovascular events, graft dysfunction, and increased mortality. Options for therapy include medical management with calcimimetic or operative management. The present study was undertaken to evaluate the natural history of HPT after renal transplantation and to determine risk factors for persistent HPT in the era of calcimimetic.

**Design:**

The study is a retrospective review of data from 74 consecutive patients who underwent renal transplantation at our institution from April 2011 to November 2019.

**Methods:**

The natural history of HPT after renal transplantation and associations between intact parathyroid hormone (PTH) level after transplantation and clinical variables such as age, sex, duration of pretransplant dialysis, and use of calcimimetic before transplantation were evaluated.

**Results:**

Intact PTH decreased after renal transplantation in most of the patients without receiving parathyroidectomy. Known risk factors of persistent HPT did not associate with intact PTH level after renal transplantation in patients who had been receiving calcimimetic before transplantation.

**Conclusion:**

In conclusion, we have found that HPT after renal transplantation could be managed successfully by medical treatments. When predicting the prognosis of HPT after transplantation, pretransplant use of calcimimetic should be taken into consideration.

## 1. Introduction

Secondary hyperparathyroidism (HPT) is a common complication in patients with chronic kidney disease. Main abnormalities responsible for secondary HPT are corrected in the first month after transplantation [1, 2]. Up to 25% of patients with secondary HPT will have persistent HPT, 1 year after transplantation [3–5]. Persistence of elevated PTH levels leads to hypercalcemia, hypophosphatemia, and reduced bone mineral density with increased fractures, as well as vascular calcification, with an increased risk of cardiovascular events, graft dysfunction, and increased mortality [6–10]. Previous studies have reported that serum calcium, alkaline phosphatase, parathyroid hormone levels at the time of transplantation, enlarged parathyroid glands detected pretransplant, and pretransplant dialysis duration are the most important risk factors for persistent HPT [3, 11–15].

Options for therapy include medical management with calcimimetic or operative management with parathyroidectomy. In Japan, cinacalcet was approved for patients with secondary HPT under maintenance dialysis in 2008 and was later indicated for severe hypercalcemia in patients with primary HPT who are unable to undergo parathyroidectomy or relapsed after parathyroidectomy and patients with parathyroid carcinoma in 2014 and evocalcet in 2018 and 2019, respectively. Etelcalcetide was approved for patients with secondary HPT under maintenance dialysis in 2020. No consensus guidelines for parathyroidectomy versus calcimimetic yet exist for persistent HPT after renal transplantation.

The use of calcimimetic may have influenced the known natural history of HPT and risk factors of persistent HPT. However, this issue remains to be elucidated. Therefore, the present study was undertaken to evaluate the natural history of HPT after renal transplantation and to determine risk factors for persistent HPT in the era of calcimimetic.

## 2. Methods

### 2.1. Study Population and Design

This study is a retrospective review of data from 74 consecutive patients who underwent renal transplantation at Tokyo Women's Medical University Hospital, from April 2011 to November 2019. Patients were excluded from study participation if they met any of the exclusion criteria: younger than 16 years, ineligibility judged by the principal investigator. The study was approved by the Ethical Review Committee of Tokyo Women's Medical University Hospital (No. 5606, May 28, 2020).

Clinical variables for analysis included age, sex, body mass index (BMI), etiology of end stage renal disease (ESRD), duration of pretransplant dialysis, cadaveric donor utilization, history of diabetes mellitus (DM) and hypertension (HTN), and smoking and drinking habits. Calcimimetic, vitamin D, bisphosphonate, and denosumab usage, total proximal femur bone mineral density (BMD), and biochemical markers including intact parathyroid hormone (PTH) (reference range: 10–65 pg/mL, electrochemiluminescence immunoassay) estimated glomerular filtration rate (eGFR), adjusted serum calcium (AdCa), and inorganic phosphate (IP) were analyzed before transplantation and at multiple posttransplant time points (12, 24, 36, 48, 60, and 72 months). In patients who were on hemodialysis before transplantation, biochemical markers were measured before dialysis. The eGFR was reported in mL/min/1.73 m^2^, using Modification of Diet in Renal Disease equation [16]. Persistent hyperparathyroidism was defined as hyperparathyroidism which needs either medication of calcimimetic, vitamin D, bisphosphonate, denosumab, and/or parathyroidectomy after renal transplantation.

### 2.2. Statistics

All statistical analyses were performed with JMP® Pro 13.0.0 software (SAS Institute Inc, Cary, NC, USA). Patient characteristics were compared and analyzed using the Wilcoxon rank sum test for continuous data, while Pearson's chi-square test was used for categorical data. The changes in variables were analyzed by one-way analysis of variance for repeated measures and for comparison of 0 month vs. and 12 months vs. each time point, Tukey's honestly significant difference test was used. Multiple linear regression was used to test the associations between intact PTH after renal transplantation and clinical variables. Multivariate logistic regression was performed to evaluate association of clinical variables with persistent hyperparathyroidism. A *p* value of <0.05 was considered significant.

## 3. Results

### 3.1. Patient Characteristics

Patient characteristics are summarized in Table 1. A total of 74 patients were included in the analysis. The median age of the entire cohort at the time of transplantation was 51 years (intraquartile range (IQR) 18). In the cohort, 49% of patients were male and median BMI was 21.5 kg/m^2^ (IQR 4.7). Etiology of ESRD was most commonly chronic glomerulonephritis (52%); type 2 DM (12%); nephrosclerosis (7%); and more rarely, type 1 diabetes mellitus, and polycystic kidney disease, among others. Patients spent a median of 3 years (IQR 11) on dialysis before renal transplantation. The type of the kidney used during transplantation was 14 cadaveric (19%) and 60 living (81%) donors. Thirty-six percent received calcimimetic (cinacalcet 25–100 mg/d or etelcalcetide 15–45 mg/wk), 74% (*n* = 55) received vitamin D (alphacalcidol, maxacalcitol, or calcitriol), and 5% (*n* = 4) received bisphosphonate before transplantation. The median pretransplant level of intact PTH was 215 pg/mL (IQR 179). Median observation period was 36 months (IQR 36).

There were no significant differences in age, sex, BMI, history of DM and HTN, smoking and alcohol habit, intact PTH, and adjusted serum calcium before transplantation between patients who had been receiving calcimimetic and not. Duration of dialysis before transplantation was significantly longer in patients who had been receiving calcimimetic (calcimimetic + vs. −, 12 years (IQR 9) vs. 1 year (IQR 3), *p* value ≤0.0001).

### 3.2. Intact PTH Level

**Figure 1:**
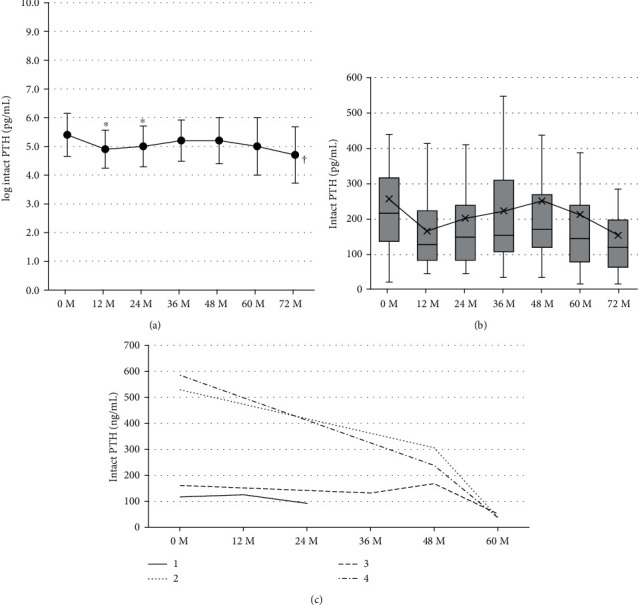
Levels of log intact PTH (mean ± SD) (a) and intact PTH (median, IQR) (b) at baseline and at 12, 24, 36, 48, 60, and 72 months following renal transplantation without parathyroidectomy and levels of intact PTH (c) at baseline and at 12, 24, 36, 48, and 60 months following renal transplantation in patients who received parathyroidectomy posttransplantation. PTH, parathyroid hormone. ^*∗*^*P* value <0.05 vs. 0 M Tukey's test. ^†^*P* value <0.05, multivariate repeated-measures approach.

Figures 1(a) and 1(b) show changes in log intact PTH level and intact PTH level after renal transplantation. Patients who received parathyroidectomy were excluded. Log PTH significantly decreased after transplantation (multivariate repeated-measures approach, *p*=0.0034, Tukey's test, 0 month vs. 12 months, 5.4 ± 0.75 pg/mL vs. 4.9 ± 0.66 pg/mL, *p*=0.0223, vs. 24 months, vs. 5.0 ± 0.71 pg/mL, *p*=0.0217). Levels of intact PTH in patients who received parathyroidectomy posttransplant (*n* = 4) are shown in Figure 1(c). PTH decreased after parathyroidectomy in all cases.

### 3.3. EGFR, AdCa, and IP

**Figure 2:**
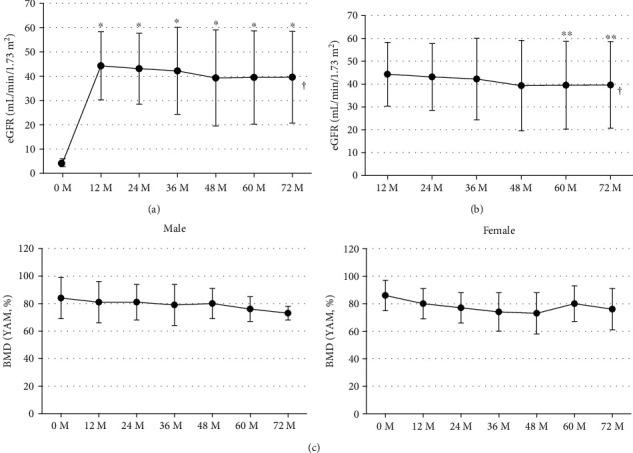
Levels of eGFR (mean ± SD) at baseline and at 12, 24, 36, 48, 60, and 72 months following renal transplantation (a) at baseline and at 12, 24, 36, 48, 60, and 72 months (b) and levels of total proximal femur BMD (mean ± SD) at baseline and at 12, 24, 36, 48, 60, and 72 months following renal transplantation by sex (c). Patients who received parathyroidectomy were excluded. eGFR, estimated glomerular filtration rate; BMD, bone mineral density. ^*∗*^*P* value < 0.05 vs. 0 M, Tukey's test. ^∗∗^*P* value <0.05 vs. 12 M, Tukey's test.

**Figure 3:**
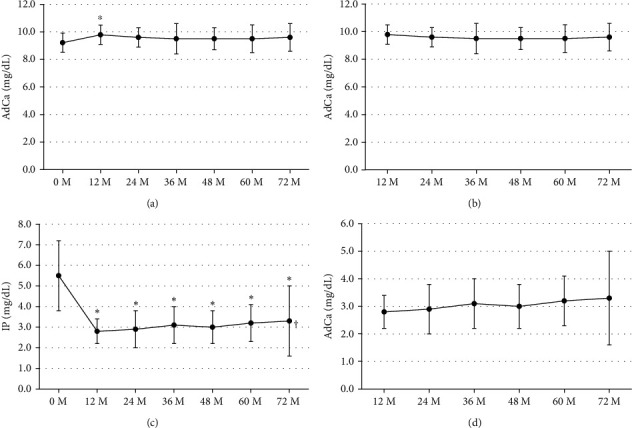
Levels of adjusted serum Ca (AdCa, mean ± SE) at baseline and at 12, 24, 36, 48, 60, and 72 months following renal transplantation (a) at 12, 24, 36, 48, 60, and 72 months (b), levels of IP (mean ± SD) at baseline and at 12, 24, 36, 48, 60, and 72 months following renal transplantation (c) at 12, 24, 36, 48, 60, and 72 months (d). Patients who received parathyroidectomy were excluded. AdCa, adjusted serum calcium; IP, inorganic phosphorus. ^*∗*^*P* value <0.05 vs. 0 M, Tukey's test. ^∗∗^*P* value <0.05 vs. 12 M, Tukey's test. ^†^*P* value <0.05, multivariate repeated-measures approach.

Changes in eGFR are shown in Figures 2(a) and 2(b). EGFR once significantly increased after transplantation (multivariate repeated-measures approach, *p* < 0.001), and it showed slight significant decrease after 12-month posttransplant (multivariate repeated-measures approach, *p* = 0.0053, Tukey's test, 12 months vs. 60 months, 44.3 ± 14.0 mL/min/1.73 m^2^ vs. 39.5 ± 19.2 mL/min/1.73 m^2^, *p*=0.0120, vs. 72 months, vs. 39.6 ± 18.9 pg/mL, *p*=0.0456). Changes in AdCa and IP are shown in Figure 3. AdCa significantly increased 12 months after transplantation (Tukey's test, 0 month vs. 12 months, 9.2 ± 0.7 mg/dL vs. 9.8 ± 0.7 mg/dL, *p*=0.0135), but remained unchanged thereafter. Also, IP significantly decreased within 12 months after transplantation (multivariate repeated-measures approach, *p* < 0.0001) and remained unchanged thereafter.

### 3.4. BMD

Figure 2(c) shows changes in total proximal femur BMD after renal transplantation by sex. There was no significant change in BMD throughout the observation period both in male and female (multivariate repeated-measures approach, male, *p*=0.7576; female, *p*=0.0679).

### 3.5. Associations between Intact PTH after Renal Transplantation and Clinical Variables

Multiple linear regression was used to test the associations between intact PTH after renal transplantation (intact PTH level at the end of each case's observation period) and clinical variables (Table 2). Duration of dialysis before renal transplantation (*p*=0.171), intact PTH before renal transplantation (*p*=0.0352), and medication of calcimimetic (*p*=0.420) is significantly positively correlated with intact PTH level after renal transplantation. The EGFR and history of parathyroidectomy after renal transplantation (*p*=0.0169) are significantly negatively correlated. There were no significant correlations between age, month after renal transplantation, and adjusted serum Ca level with the intact PTH level after renal transplantation.

Subgroup analysis on associations between the intact PTH level after renal transplantation and clinical variables by with/without medication of calcimimetic before transplantation was done (Table 3). There were no significant associations between duration of dialysis, level of intact PTH pretransplantation, and eGFR and level of intact PTH posttransplantation in patients who had been receiving calcimimetic before transplantation.

### 3.6. Predictors for Persistent Hyperparathyroidism

Table 4 provides odds ratios for the association of each variable within the predictive model to persistent hyperparathyroidism. Duration of dialysis pretransplantation is significantly related to the risk of persistent hyperparathyroidism (odds ratio: 5.1, *p* = 0.0033).

### 3.7. Percentage of Patients Treated with Medications Affecting Bone and Mineral Metabolism

**Figure 4:**
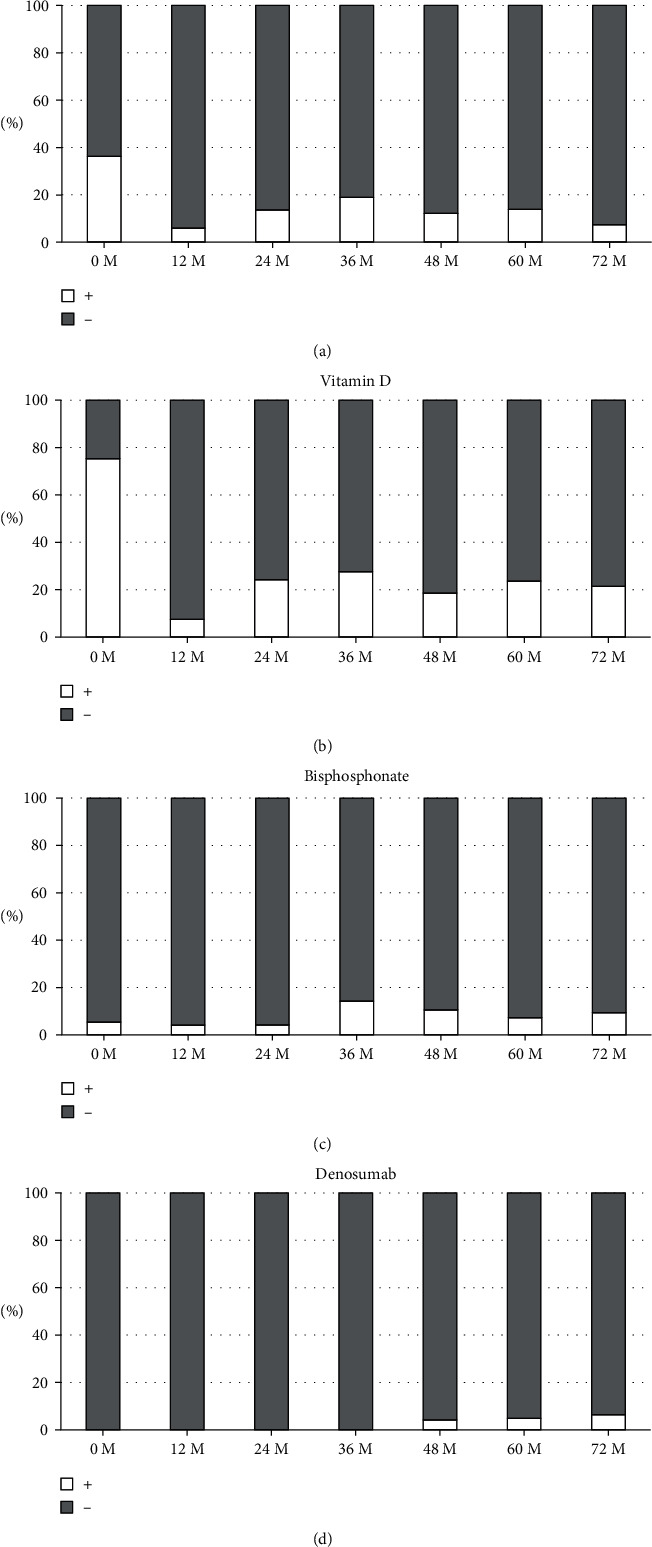
Percentage bar graph of patients treated with calcimimetic (a), vitamin D (b), bisphosphonate (c), and denosumab (d).

Figure 4 shows percentage bar graph of patients treated with calcimimetic, vitamin D, bisphosphonate, and denosumab. Treatment with calcimimetic and vitamin D decreased after renal transplantation (calcimimetic: 0 M, 36%; 12 M, 6%; 72 M, 7%; vitamin D: 0 M, 75%; 12 M, 7%; and 72 M, 21%). Treatment with bisphosphonate and denosumab tended to increase after transplantation.

## 4. Discussion

The present study demonstrated two major findings. First, intact PTH decreased after renal transplantation in most of the patients without receiving parathyroidectomy. Second, long duration of dialysis and elevated intact PTH level before transplantation and posttransplant decreased eGFR, known risk factors of persistent HPT, did not associate with intact PTH level after renal transplantation in patients who had been receiving calcimimetic before transplantation.

Mean renal allograft eGFR at the end of the observational period was 41.0 ± 17.5 mL/min/1.73 m^2^ in our cohort. In patients with chronic kidney disease G3a–G5 not on dialysis, the optimal intact PTH level is not known [17]. In the present study, except for 4 patients who needed to have parathyroidectomy, the intact PTH level significantly decreased without performing parathyroidectomy but persisted above the upper normal limit. Prolonged HPT has been known to be associated with hypercalcemia and hypophosphatemia, but serum AdCa and IP were controlled to normal range after transplantation in our study. Pihlstrøm et al. found that PTH above the upper limit of normal indicated 85% higher risk of graft loss compared with low/normal values [6]. Finnerty et al. also demonstrated that performing parathyroidectomy for persistent HPT was associated with improved renal allograft function as compared with cinacalcet [18]. However, in patients managed medically with cinacalcet alone, renal allograft failure was associated with increased PTH within 12 months posttransplant compared with patients with a functioning allograft (348 pg/mL vs. 195 pg/mL) [18]. Median PTH level in our subject (who did not receive parathyroidectomy) at 12-month posttransplant was 165 pg/mL (IQR 140), with 11 patients receiving calcimimetic and 63 patients not. Also, though allograft renal function slightly decreased, the decrease was comparable to that of Japanese general population with an initial GFR of 40–49 mL/min/1.73 m^2^ [19]. Heaf et al. reported that in patients with the high intact PTH level (>150 ng/L) after transplantation, although lumbar spine BMD stabilized after renal transplantation, there was a continuous loss of femoral neck BMD [20]. Total proximal femur BMD in our study remained unchanged. Overall, these evidences indicate that HPT after renal transplantation could be successfully managed without performing parathyroidectomy, regarding allograft renal function and BMD.

We have analyzed the associations between the intact PTH level after renal transplantation (intact PTH level at the end of each case's observational period) and clinical variables. Consistent with the previous reports, duration of dialysis before renal transplantation and intact PTH before renal transplantation were risk factors for the increased intact PTH level after RT. Contrary to our expectation, medication of calcimimetic was also an independent risk factor for the persistence of HPT. It is speculated that this was because in the first place, calcimimetic was given selectively to patients with higher persistent PTH level. Conversely, having parathyroidectomy is negatively correlated with posttransplant PTH level. This might indicate that parathyroidectomy is superior to calcimimetic with respect to lowering PTH level.

Subgroup analysis showed no significant associations between duration of dialysis, level of intact PTH pretransplantation, and eGFR and level of intact PTH posttransplantation, in patients who had been receiving calcimimetic before transplantation. Actually, though calcimimetic is not covered by insurance for persistent HPT after renal transplantation in Japan, it is used in clinical practice, and we observed the course retrospectively. The variables known to be the most important risk factors for tertiary HPT [3, 11–15] lost relevance in the group with calcimimetic. Duration of dialysis lost relevance both in the groups with and without calcimimetic. Since the duration was significantly longer in the group with calcimimetic, within-group variance might have been too small for the duration of dialysis to be an independent risk for tertiary HPT in each subgroup. Level of intact PTH pretransplantation was not an independent risk factor in the group with calcimimetic. Although the intact PTH level had been successfully decreased by calcimimetic before transplantation in patients with HPT, it might not have cured HPT. It is speculated that since most of the patients discontinued receiving calcimimetic after transplantation, masked HPT manifested after transplantation and pretransplant and posttransplant level of PTH are no longer associated.

This study has several limitations. The first is that it was based on retrospective data analysis. Although prospective validation would be ideal, since all the variables evaluated were objective clinical information and we also had a high rate of complete data collection, it is unlikely that the results would greatly change even if analyzed prospectively. Furthermore, because of the size limitation of the study, we were unable to include adequate factors in the multivariate analyses, particularly in subgroup analysis. Also, since it was a single-center study, selection of medication for HPT might have been biased. Finally, since we defined persistent HPT as HPT which needed either medication of calcimimetic, vitamin D, bisphosphonate, denosumab, and/or parathyroidectomy after renal transplantation, two different states with different pathophysiology, secondary, and tertiary HPT might have been mixed in our cohort. To fully explore the influence of calcimimetic, given before transplantation, on persistent HPT, implementation of a prospective, randomized controlled study is needed.

In conclusion, we have found that HPT after renal transplantation could be managed without surgical treatment in the era of calcimimetic. When predicting the prognosis of HPT after transplantation, pretransplant use of calcimimetic should be taken into consideration.

## Figures and Tables

**Table 1:** Patient characteristics before RT.

	All		Calcimimetic		*P* value
			With	Without	
*n*	74		27	47	
Age, year	51 (18)		54 (16)	46 (21)	0.0703
Male, *n*	36 (49)		13 (48)	23 (49)	0.9479
BMI, kg/m^2^	21.5 (4.7)		21.3 (3.3)	21.5 (5.0)	0.7026
Underlying disease, *n*	IgA nephropathy	13 (18)			
	Other CGN	25 (34)			
	Type 1 DM	4 (5)			
	Type 2 DM	9 (12)			
	Nephrosclerosis	5 (7)			
	Others	15 (20)			
	Unknown	3 (4)			
Duration of dialysis pretransplantation, year	3 (11)		12 (9)	1 (3)	**<0.0001**
Cadaveric donor kidney transplant, *n*	14 (19)		9 (33)	5 (11)	**0.0184**
History of DM, *n*	15 (20)		5 (19)	10 (21)	0.7753
History of HTN, *n*	46 (62)		16 (59)	30 (64)	0.6969
Smoking, *n*	17 (23)		4 (15)	13 (28)	0.1950
Alcohol, *n*	24 (33)		9 (33)	15 (32)	0.9002
Calcimimetic medication, *n*	27 (36)				
Vitamin D medication, *n*	55 (74)		23 (85)	32 (68)	0.0949
Bisphosphonate medication, *n*	4 (5)		3 (11)	1 (2)	0.1065
Denosumab medication, *n*	0 (0)		0 (0)	0 (0)	
BMD (YAM), %	87 (16)		88 (19)	86 (20)	0.5584
Intact PTH, pg/mL	215 (179)		209 (159)	241 (20)	0.6901
eGFR, ml/min/1.73 m^2^	3.9 (1.6)		3.4 (0.7)	4.4 (2.4)	**0.0013**
Adjusted ca, mg/dL	9.0 (1.0)		9.4 (0.6)	9.0 (0.9)	0.1193
IP, mg/dL	5.4 (2.1)		5.5 (1.7)	5.2 (2.7)	0.7492

Data are median (IQR, intraquartile range) for continuous variables and number (percent) for categorical variables. Bold values are those found to be significant; *p* value <0.05. RT, renal transplantation; BMI, body mass index; DM, diabetes mellitus; HTN, hypertension; BMD, bone mineral density; YAM, young adult mean; PTH, parathyroid hormone; eGFR, estimated glomerular filtration rate.

**Table 2:** Associations between intact PTH after RT and clinical variables.

Covariates	log intact PTH, ng/mL	Standardized *β*	*P* value
Intercept	5.305	0	**<0.0001**
Age, year	−0.009	−0.13	0.2821
Duration of dialysis pretransplantation, year	0.003	0.32	**0.0171**
Log intact PTH before RT	0.274	0.24	**0.0352**
Month after RT	−0.003	−0.09	0.3951
eGFR	−0.018	−0.35	**0.0082**
Adjusted serum Ca	−0.096	−0.12	0.3414
Parathyroidectomy	−0.438	−0.29	**0.0169**
Medication of calcimimetics	0.227	0.23	**0.0420**

The data are the ng/mL associated with each listed covariate, after adjustment for all other covariates listed. The intercept value is added to the covariate value (after appropriate weighting) to obtain the intact PTH estimate. For parathyroidectomy, the intact PTH level in patient who underwent parathyroidectomy compared with patient who did not undergo parathyroidectomy is shown. For medication of calcimimetic, the intact PTH level in patient with medication of calcimimetic compared with patient without medication of calcimimetic is shown. Bold values are those found to be significant; *p* value <0.05. PTH, parathyroid hormone; RT, renal transplantation; eGFR, estimated glomerular filtration rate.

**Table 3:** Associations between intact PTH after RT and clinical variables by with/without medication of calcimimetic.

	Calcimimetic					
	With			Without		
Covariates	Log intact PTH, ng/mL	Standardized *β*	*P* value	Log intact PTH, ng/mL	Standardized *β*	*P* value
Intercept	4.599	0	**0.0001**	3.930	0	**<0.0001**
Duration of dialysis pretransplantation, year	−0.003	−0.05	0.8337	0.004	0.03	0.8177
Log intact PTH before RT	0.192	0.24	0.3035	0.362	0.33	**0.0159**
eGFR	−0.006	−0.23	0.3228	−0.028	−0.55	**0.0002**

The data are given in ng/mL associated with each listed covariate, after adjustment for all other covariates listed. The intercept value is added to the covariate value (after appropriate weighting) to obtain the intact PTH estimate. Bold values are those found to be significant; *p* value <0.05. PTH, parathyroid hormone; RT, renal transplantation; eGFR, estimated glomerular filtration rate.

**Table 4:** Predictors for persistent hyperparathyroidism.

Characteristic	Odds ratio (95% CI)	*P* value
Age		
<51 y	Reference	—
≥51 y	1.3 (0.5–3.8)	0.5526

Duration of dialysis pretransplantation		
<3 y	Reference	—
≥3 y	5.1(1.7–15.2)	**0.0033**

PTH before		
<215 ng/mL	Reference	—
≥215 ng/mL	1.2 (0.4–3.3)	0.7581

Persistent hyperparathyroidism includes hyperparathyroidism which needed either medication of calcimimetic, vitamin D, bisphosphonate, denosumab, and/or parathyroidectomy after renal transplantation. Bold values are those found to be significant; *p* value < 0.05. RT, renal transplantation; PTH, parathyroid hormone.

## Data Availability

The data used to support the findings of this study are included within the article.
